# L718Q/V mutation in exon 18 of EGFR mediates resistance to osimertinib: clinical features and treatment

**DOI:** 10.1007/s12672-022-00537-7

**Published:** 2022-08-09

**Authors:** Meihui Li, Jing Qin, Fajun Xie, Lei Gong, Na Han, Hongyang Lu

**Affiliations:** 1 Zhejiang Key Laboratory of Diagnosis & Treatment Technology On Thoracic Oncology (Lung and Esophagus), Cancer Hospital of the University of Chinese Academy of Sciences (Zhejiang Cancer Hospital), 310022 Hangzhou, P. R. China; 2 Department of Thoracic Medical Oncology, Cancer Hospital of the University of Chinese Academy of Sciences (Zhejiang Cancer Hospital), 310022 Hangzhou, P. R. China; 3 Institute of Basic Medicine and Cancer (IBMC), Chinese Academy of Sciences, 310022 Hangzhou, P.R. China; 4 The First Clinical Medical College, Wenzhou Medical University, 325035 Wenzhou, P. R. China

**Keywords:** L718Q/V mutation of EGFR exon 18, EGFR T790M mutation, Osimertinib, Resistance mechanism, Afatinib

## Abstract

Osimertinib, a mutant-specific third-generation epidermal growth factor receptor (EGFR) tyrosine kinase inhibitor (TKI), is emerging as the preferred first-line of treatment for EGFR-mutant lung cancer. However, osimertinib resistance inevitably develops among patients treated with the drug. The modal resistance mechanisms of osimertinib include the occurrence of epithelial transition factor (c-MET) amplification and C797S mutation, whereas rare mutations are presented as case reports. Recently, the L718Q/V mutation in exon 18 of EGFR has been reported to contribute to one of the possible mechanisms of resistance. The clinical features and subsequent treatment strategies for this mutation require further research. This study retrospectively enrolled NSCLC patients with the L718Q/V mutation from 2017 to 2021 at the Cancer Hospital of the University of the Chinese Academy of Sciences (Zhejiang Cancer Hospital), as well as additional patients with the same mutation from PubMed literature, to summarize the clinical features of the mutation. The association between the detection of L718Q/V and resistance to osimertinib, as well as impacts on the therapeutic process and outcome, was analyzed. We included a total of two patients diagnosed at Zhejiang Cancer Hospital and twelve patients from the literature. Of the fourteen total patients, 64.3% were male and 35.7% were female. The average age of the group was 60.2 years (range 45–72). A history of tobacco use was common among the group. In all of the cases we considered, the L718Q/V mutation was secondary to the L858R mutation. The second-generation TKI afatinib was found to provide a high disease control rate (DCR) (85.7%, 6/7) and relatively low objective response rate (ORR) (42/9%, 3/7). The median progression free survival (mPFS) for this treatment reached 2 months (1–6 months). The patients failed to benefit from chemotherapy combined with immunotherapy or other TKI medications. Due to the limited number of cases considered in this study, future studies should explore drugs that more precisely target the L718Q/V mutation of EGFR exon 18.

## Introduction

The strategy for treating non-small cell lung cancer (NSCLC) has drastically changed over the past few decades, shifting from traditional therapy including surgery, radiotherapy, and chemotherapy to newer classes of treatment such as target-therapy and immunotherapy. Many large-scale clinical trials have demonstrated that targeted drugs, such as epidermal growth factor receptor (EGFR) tyrosine kinase inhibitors (TKIs), have become a major and preferred treatment modality for advanced NSCLC with EGFR mutations. Among these medications, the third-generation targeted drug osimertinib has resulted in significant improvements in progression free survival (PFS) and overall survival (OS), both in the second-line treatment of patients with acquired T790M mutations [1, 2] and the first-line treatment of locally advanced or metastatic EGFR-mutant NSCLC [3, 4]. Nevertheless, the development of osimertinib resistance among these patients has been a gradual and inevitable trend, limiting the median PFS to 10.1 months [1, 5, 6]. The mechanisms of osimertinib resistance can be roughly divided into the following two classes: the EGFR-related drug resistance mechanism, and the EGFR-unrelated drug resistance mechanism, and so on [7]. The stage of cancer and type of genetic mutation have been found to be associated with drug resistance, as especially evident in the mechanism of osimertinib resistance. It is prospective to explore the drug resistance mechanism after the application of osimertinib and find out the effective drugs.

In this paper, we retrospectively analyzed two cases of lung adenocarcinoma with L718Q/V mutations of EGFR exon 18 through next generation sequencing (NGS) technique. The L718 residue of the EGFR exon 18 is located at the ATP binding site of the EGFR kinase domain inducing the L718Q and L718V mutations [7]. After reviewing the therapeutic history for both patients, we speculated that the presence of the L718Q/V mutation in EGFR exon 18 may contribute to the acquired resistance mechanism of the EGFR T790M mutation. Furthermore, we integrated previous reports on treatments involving the L718Q/V mutation into our discussion to explore the most effective therapeutic strategies that may be used after the occurrence of this mutation.

## Materials and methods

### Patient and clinical sample collection

A comprehensive search was performed through PubMed using the literature retrieval strategy “[EGFR L718Q] OR [EGFR L718V] AND [Osimeitinib (Title/Abstract)]” in December 2021 (no year limit and all languages included). Relevant articles were obtained, and references from each of these articles were further searched for relevant articles. A total of 14 articles were reviewed (case reports or case series) and 12 patients with definite molecular pathological diagnosis were collected. Two patients confirming EGFR L718Q/V in Cancer Hospital of the University of Chinese Academy of Sciences (Zhejiang Cancer Hospital) were enrolled with a history of EGFR-TKIs application based on patient's genetic testing results.

### DNA extraction and NGS

DNA was extracted from tumor tissue, plasma, and cerebrospinal fluid. Library preparation and quantification were carried out. Then, we performed hybridization based on targeted enrichment and sequencing. Briefly, circulating tumor DNA (ctDNA) was extracted using the ATG-Seq technique (liquid biopsy system) by following an optimized version of the manufacturer’s protocols. The ctDNA was examined for 139 lung cancer-related genes based on the LungTrak panel, which includes diverse Chinese data and the well-regarded TCGA database. The results of this analysis included mutations related to lung cancer (point mutations and small fragment insertion-deletion mutations), gene fusion and copy number variations, microsatellite (MS) analysis, and tumor mutant load (TMB).

### Curative effect judgment

Patient’s response to therapies and the progression of different metastatic lung lesions were monitored by computed tomography (CT) imaging and clinical symptoms plus laboratory inspections. The response evaluation was assessed according to the Response Evaluation Criteria in Solid Tumors (RECIST, version 1.1) including complete response (CR), partial response (PR), stable disease (SD), and progressive disease (PD) [8]. The OS and PFS were used as the observation indexes.

### Follow-up

The follow-up deadline was December 31, 2021. Two patients were deceased. The survival time was counted from the date of pathological diagnosis.

## Results

### Overview of the patients

Of the fourteen total patients with L718Q/V mutations in exon 18 of EGFR that were enrolled in our study, two were diagnosed at our hospital and twelve were from PubMed literature. The basic clinical features of the patients are classified in Table 1*.* Nine (64.3%) of the patients were male and five (35.7%) were female. The study group was composed of middle aged and elderly individuals, with an average age of 60.2 years and age range of 45–72 years. Nine of the patients (64.3%) were smokers. At the time of diagnosis, six of the patients (42.9%) had stage IV cancer, one (7.1%) had stage IIB cancer, and the stage of the remaining seven patients (50%) was unknown.

**Table 1:** Clinical characteristics of patients with L718Q/V mutations of EGFR exon 18

References	Sex	Age	Smoking	Lesion	Stage	Original mutation
Case1	Male	62	Yes	Right	IV	L858R
Case2	Male	67	Yes	Right	IV	L858R
Case3 [15]	Male	59	Yes	un	IV	L858R
Case4 [17]	Male	67	Yes	Left	IIB	L858R
Case5 [18]	Female	62	Yes	Right	IV	L858R
Case6 [21]	Male	58	Yes	un	un	L858R
Case7 [21]	Female	72	No	un	un	L858R
Case8 [21]	Male	45	Yes	un	un	L858R
Case9 [22]	Female	65	No	Right	IV	L858R
Case10 [23]	Male	45	Yes	Left	IV	L858R
Case11 [24]	Female	un	No	un	un	L858R
Case12 [24]	Female	un	No	un	un	L858R
Case13 [24]	Male	un	Yes	un	un	L858R
Case14 [24]	Male	un	No	un	un	L858R

un, unknown

### NGS

The L718V mutation was not originally detected in liquid or tissue biopsies taken at the time of diagnosis for any of the patients prior to starting osimertinib therapy. Additionally, the original mutation present in all patients was L858R in EGFR exon 21. None of the patients developed alterations in EGFR C797S resistance or any other molecular mechanisms of resistance, at the time of progression on osimertinib.

### Treatment process of the two cases

**Fig. 1:**
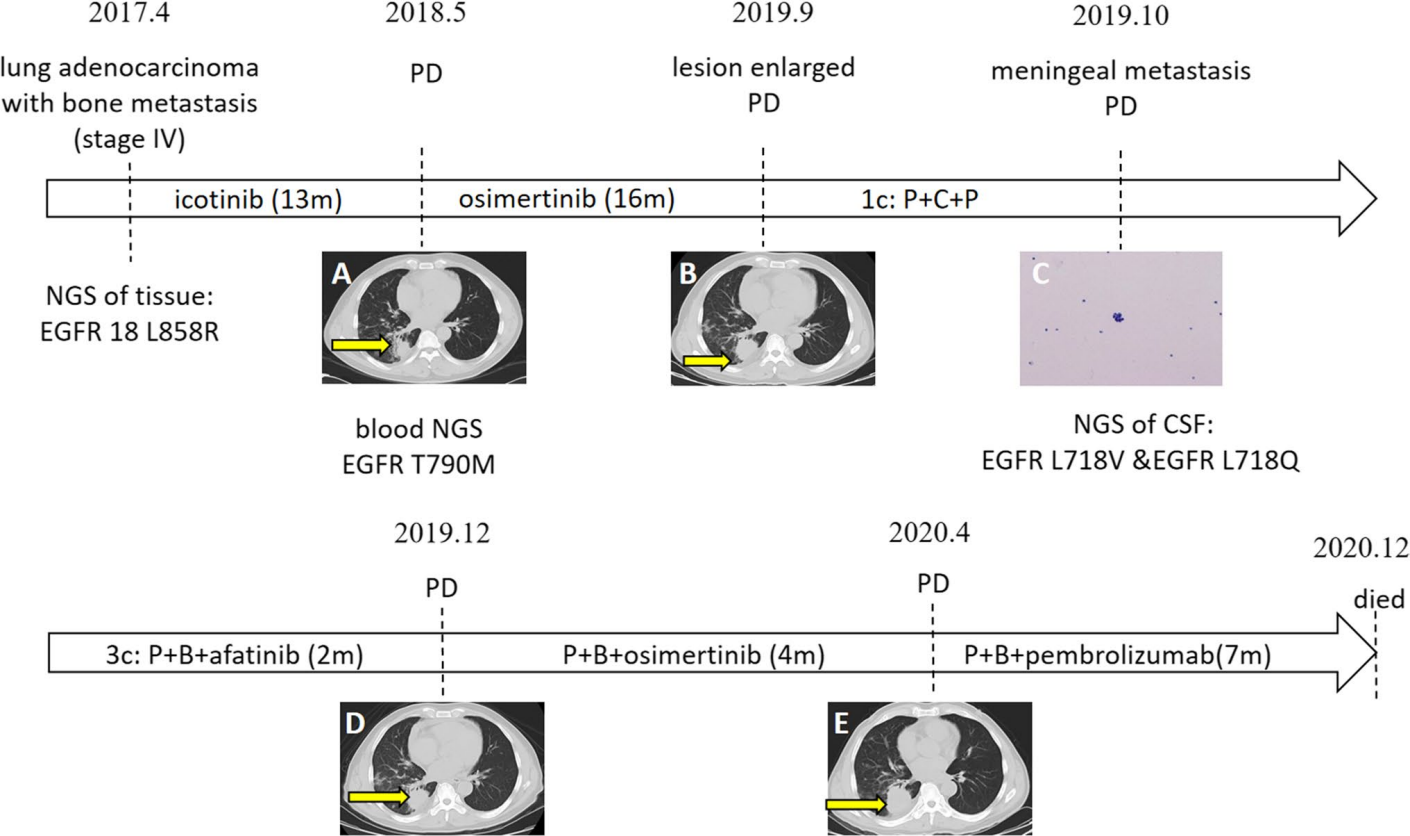
Detailed diagnosis and treatment procedures of Case1. The patient complained of chest tightness and shortness of breath after going upstairs and was diagnosed with lung adenocarcinoma and bone metastasis (stage IV) at other medical centers in April 2017. L858R mutation in exon 21 of EGFR was revealed by lung puncture biopsy. Icotinib was applied for 13 months, and the best curative effect was partial response (PR). The concentration of serum CEA and re-examination in May 2018 via computerized tomography (CT) showed disease progression (PD). The mutation was confirmed to be EGFR T790M through genetic testing of the blood, since the patient was unwilling to undergo another biopsy (**A**). Osimertinib was administered daily for 16 months and PR was achieved with good physical condition. CT images in September 2019 showed PD (**B**). Pemetrexed (500 mg/m^2^) combined with carboplatin (area under the curve ¼ 5) plus pembrolizumab (200 mg) was administered only once since an unexpected syncope before the second treatment on October 8, 2019. Meningeal metastasis was diagnosed by adenocarcinoma cells found in the cerebrospinal fluid (CSF) obtained from a lumbar puncture (**C**) and enhanced magnetic resonance image (MRI). Genetic testing indicated the presence of the L718V (c.2152C > G (p.L718V), 7.7%) and L718Q (c.2153 T > A (p.L718Q), 1.6%) mutations of EGFR exon 18. Pemetrexed combined with bevacizumab (15 mg/kg) plus afatinib was administered for three cycles, and the best response achieved stable disease (SD). CT performed in December 2019 showed PD (**D**). Subsequently, pemetrexed in combination with bevacizumab (15 mg/kg) plus osimertinib was applied for four months in which myelosuppression occurs. Due to progression in April 2020 (**E**), the patient was treated with pemetrexed combined with bevacizumab plus pembrolizumab until November 2020 on account of intolerance to any kind of treatment. The patient died of cachexia on December 18, 2020

**Fig. 2:**
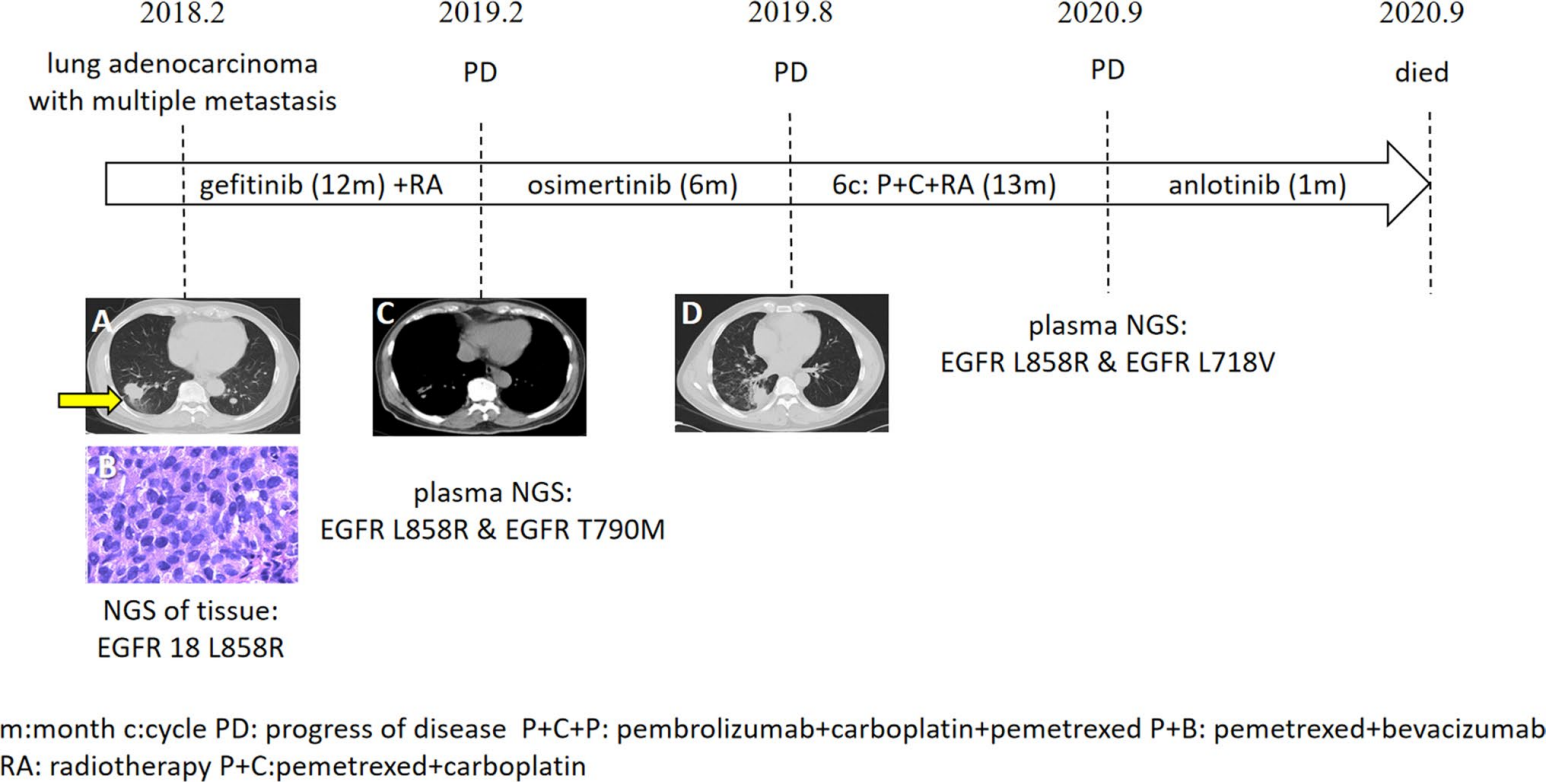
Detailed diagnosis and treatment procedures of Case2. The patient was revealed a lung occupying lesion through health examination on, and February 6, 2018. The primary tumor located in the right lower lung was punctured under CT guidance (**A**), and the pathologic diagnosis was adenocarcinoma (**B**) with multi bone metastasis. The next generation sequencing (NGS) results of the tissue indicated the presence of the L858R mutation of EGFR exon 21. Gefitinib was administered in combination with radiotherapy to the left scapula. The symptoms of ostalgia and thoracalgia rapidly improved, and the best response achieved was PR. CT re-examination in February 2019 indicated PD status (**C**) in focus of lung and bone. Lumbar disc arthroplasty was performed to protect the lumbar spine and relieve pain. In the wake of the presence of EGFR T790M mutation (c.2369C > T(p.T790M), 2.0%) plus EGFR L858R mutation (c.2573 T > G(p.L858R), 5.2%) detected by NGS of the plasma, Osimertinib was subsequently applied for six months. In August 2019, CT once again indicated PD status (**D**) and pemetrexed plus carboplatin was applied for six cycles in combination with radiotherapy to left hip. The best efficacy assessment achieved PR. In view of the progressed bone metastases and the significantly increased serum concentration of tumor markers in September 2020, plasma NGS was performed, revealing the presence of the EGFR L718V mutation (C.2152C > G(p.L718V), 13.9%). The patient was treated with anlotinib and died on September 20, 2020

Two cases involving L718Q mutations were followed as part of this study. Flow charts for the diagnosis and treatment of these patients are shown in Figs. 1 and 2. The first case describes a 62-year-old male patient who complained of shortness of breath in March of 2017. This patient was diagnosed with lung adenocarcinoma and bone metastasis (cT2N1M1, stage IV, L858R mutation in EGFR exon 21) and was treated accordingly with icotinib. A pulmonary biopsy revealed a T790M mutation in EGFR, which led to the initiation of treatment with osimertinib. The patient was classified with progressive disease (PD) status, and the combination of immunotherapy and chemotherapy was applied for one cycle. Subsequently, the L718V and L718Q mutations in exon 18 of EGFR were found from NGS of the cerebrospinal fluid (CSF). Afatinib was added to the current medications for three cycles, and the best curative effect achieved stable disease (SD) status. Later, afatinib was replaced with osimertinib for four months due to PD status. The addition of pemetrexed on the basis of immunotherapy and targeted therapy resulted in seven months of PFS. The patient died of PD on December 18, 2020.

The second case follows a patient diagnosed with lung adenocarcinoma with multiple metastases (cT2N0M1, stage IV, L858R mutation in exon 21 of EGFR) in February of 2018. Gefitinib and radiotherapy were applied as treatment, resulting in rapid and long-term improvement. After the T790M mutation in exon 20 of EGFR was discovered, treatment with osimertinib was initiated. The combination of chemotherapy and radiotherapy was employed for six cycles due to PD status. Later, a plasma biopsy revealed the appearance of the L718V mutation in EGFR, and anlotinib was subsequently added into the treatment scheme. However, the patient died less than 1 month later.

### Patient outcomes and survival

**Fig. 3:**
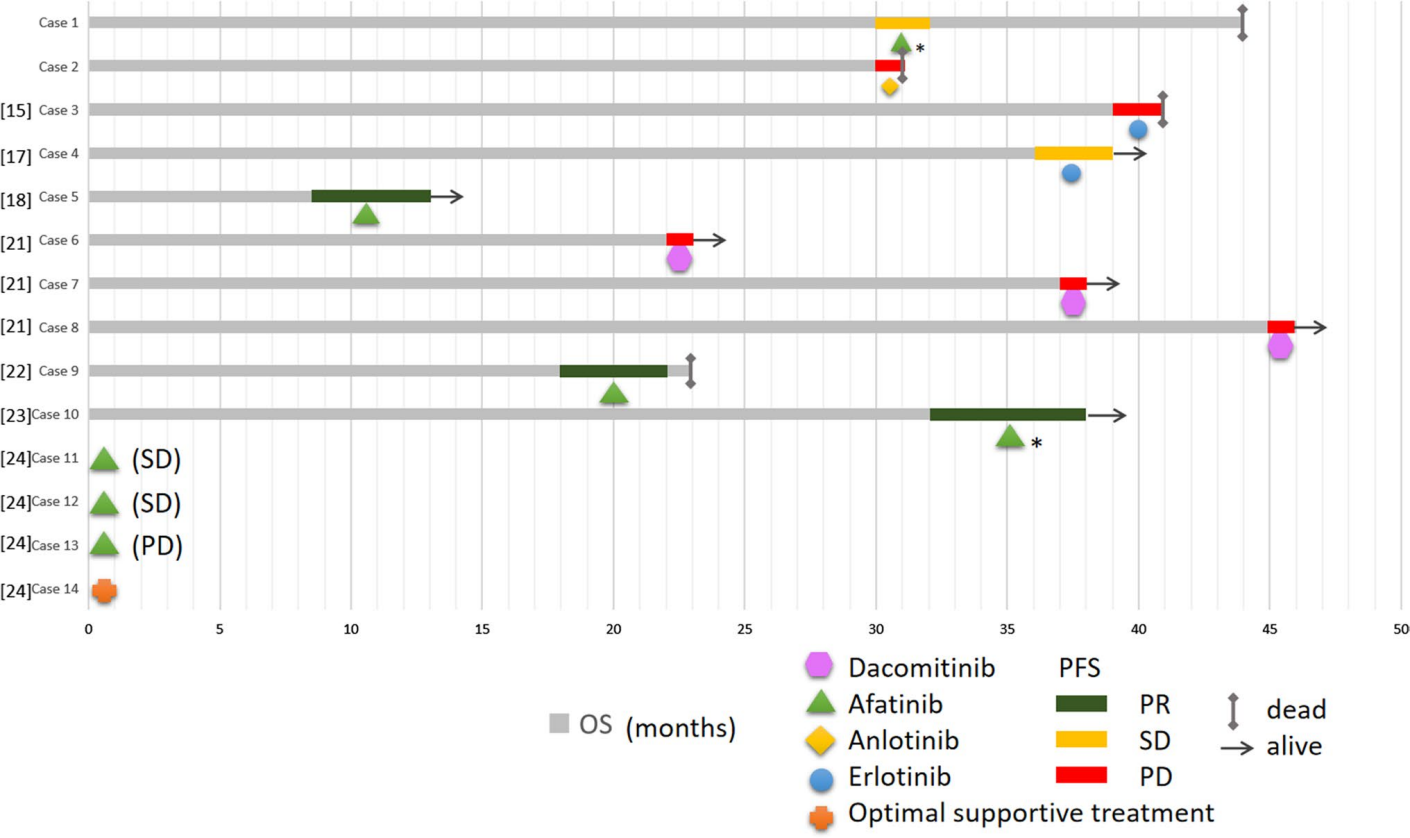
Medication to the L718Q/V mutation in exon 18 of EGFR and prognosis (OS and PFS) of ten patients. Overall survival (OS) is represented by the gray line segments, whereas progression free survival (PFS) is represented by the colorful line segments. Each grid represents a month. * stands for targeted therapy in combination with other treatments, such as chemotherapy or radiation. The PFS and OS for cases 11–14 are not shown in the figure because there was no relevant data in the literature

The two patients at our center had died by the time of the follow-up deadline. With the application of EGFR-TKIs against the L858R mutation, the patients gained 5–27 months of PFS (mean: 13.3 months; median: 12 months). Of the fourteen total patients, seven received afatinib, three received dacomitinib, two received erlotinib, one received anlotinib, and one received best supportive care (BSC) due to poor performance status (PS) as the follow-up treatment. The incidence of the L718Q/V mutation resulted in an average of 2.6 months of PFS (range 1–6 months). Three (21.4%) achieved partial remission (PR), four (28.6%) achieved SD, and six (42.9%) achieved PD. The objective remission rate (ORR) for this group was 21.4% (3/14). Of the seven patients who received afatinib, two were simultaneously undergoing other therapies such as radiotherapy, chemotherapy, and immunotherapy. The disease control rate (DCR) for the afatinib treatment group was 85.7% (6/7), and the ORR 42.9% (3/7). Of the three patients who received dacomitinib as subsequent therapy, PFS of each patient was 1 month. Overall survival (OS) ranged from 23 to 46 months. After the emergence of the L718Q/V mutation, OS ranged from 1 to 14 months. The treatments and prognoses of the patients are summarized in Fig. 3.

## Discussion

According to previous researches, the median PFS of osimertinib for the T790M mutation is 10.1 months [1, 5, 6], and majority of patients develop resistance to the drug within one year of therapy onset [9, 10]. Exploring the mechanism of osimertinib resistance may prolong its period of effective action, and therefore bring benefits to patients. In recent years, the potential mechanisms of resistance to osimertinib have been explored that research has shown that osimertinib resistance may involve acquired mutations in EGFR-independent c-MET amplification [7] and EGFR-dependent C797S [11], or rare mutations at positions P596L [1], L792 [12], G796 [13], and G724S [14]. In this study, we followed two patients with EGFR T790M mutations. These patients were treated with osimertinib, and acquired resistance less than half a year after initiating treatment. These patients also developed L718Q/V mutations in exon 18 of EGFR. Therefore, we speculate that the L718Q/V mutation in exon 18 of EGFR contributes to one of the mechanisms of acquired resistance to osimertinib. Previous studies have revealed that the L718Q mutation in EGFR, with or without the T790M mutation, accounts for approximately 2% of all patients with osimertinib resistance [5, 6, 15]. The L718 residue is located at the ATP binding site of the EGFR kinase domain, where mutations may lead to spatial structural variation and further block the binding of osimertinib to EGFR [7]. The relationship between L718Q/V and osimertinib has been studied in some previous reports. Zhu SJ [16] proved that L718V is a missense mutation that occurs in an ATP binding hydrophobic clamp region. It is hypothesized that mutations of the hydrophobic clamp may affect the binding affinity of any inhibitor that interacts with it. Yang Z et al. [15] found that the L718Q mutation in EGFR conferred the highest resistance to osimertinib by affecting the conformation of the EGFR-osimertinib complex, thus preventing reaction with C797 in vitro. Liu et al. [17] demonstrated that in vitro cells expressing EGFR L858R/L718V were able to grow better compared to those expressing L858R, it means L718V is oncogenic and renders growth advantage. The researchers also employed in silico protein structure modeling and found that L718V likely introduces a spatial confliction that prevents osimertinib from binding to EGFR.

According to the 14 patients we examined, the L718Q/V mutation was more likely to occur in middle to elderly aged male NSCLC patients with a history of tobacco use. Furthermore, this mutation did not seem specific to any pulmonary lesion location. Until now, patients with the L718V/Q mutation did not have specific and effective treatment guidelines and had to try different experimental medications. A study of transgenic murine models found that the L718Q/V mutation occurs almost exclusively in the setting of an L858R driver mutation, which was consistent with the cases examined in this paper [18]. Furthermore, in vivo models were found to show sensitivity to afatinib while demonstrating resistance to gefitinib, erlotinib, and osimertinib. Ercan [19] proved that in the presence of an EGFR-activating mutation alone, afatinib and a novel TKI in development (CL-387785) were particularly effective at inhibiting the growth of both L718Q- and L844V-mutant cells, and consequently inhibited EGFR phosphorylation and activity. The above diverse drug susceptibilities were also applied to the L718V mutation of EGFR exon 18, but the accuracy of this conclusion needs to be verified due to the rare occurrence rate of the mutation. After the occurrence of L718Q/V, the PFS for the 14 patients included in this study ranged from 1 to 6 months, and the OS ranged from 1 to 14 months. In a retrospective study which applied afatinib to non-classical EGFR mutations, the ORR was 65.2% (n = 75) [20]. The ORR was 42.9% (3/7) with a high DCR in the patients treated with afatinib that we studied, the rapid progression may be attributed to the fact that the patients were all treated in the posterior line or the cachexia. In the future, more intensive clinical experimental studies are needed to prove the efficacy of afatinib on account of the rarity of cases. Other second-generation EGFR-TKI Dacomitinib may be ineffective in patients with L718Q/V mutation providing PFS of only 1 months in three patient [21]. In addition, there is currently no relevant research that explores whether the occurrence of L718Q/V will affect the prognosis of the patient – in other words, future research should investigate whether the mutation is a marker of poor prognosis.

## Conclusion

The L718Q/V mutation in exon 18 of EGFR may contribute to the mechanism of osimertinib resistance. Until now, there has been no recommended treatment guidance for this mutation. Future clinical studies should apply different medications in more intensive experimental studies to better understand the optimal treatment strategy. Although clinical trials on the various mechanisms of drug resistance have been conducted, some clinical applications are still far from effective. Therefore, the primary future research directions of oncological medicine should focus on exploring potential drug resistance mechanisms and more reasonable strategies of drug timing.

## Data Availability

The datasets generated during and/or analysed during the current study are available from the corresponding author on reasonable request.
